# Angiotensin II and the Cardiac Parasympathetic Nervous System in Hypertension

**DOI:** 10.3390/ijms222212305

**Published:** 2021-11-14

**Authors:** Julia Shanks, Rohit Ramchandra

**Affiliations:** Department of Physiology, University of Auckland, Auckland 1023, New Zealand; julia.shanks@auckland.ac.nz

**Keywords:** Ang II, autonomic nervous system, parasympathetic activity, cardiac vagal activity

## Abstract

The renin–angiotensin–aldosterone system (RAAS) impacts cardiovascular homeostasis via direct actions on peripheral blood vessels and via modulation of the autonomic nervous system. To date, research has primarily focused on the actions of the RAAS on the sympathetic nervous system. Here, we review the critical role of the RAAS on parasympathetic nerve function during normal physiology and its role in cardiovascular disease, focusing on hypertension. Angiotensin (Ang) II receptors are present throughout the parasympathetic nerves and can modulate vagal activity via actions at the level of the nerve endings as well as via the circumventricular organs and as a neuromodulator acting within brain regions. There is tonic inhibition of cardiac vagal tone by endogenous Ang II. We review the actions of Ang II via peripheral nerve endings as well as via central actions on brain regions. We review the evidence that Ang II modulates arterial baroreflex function and examine the pathways via which Ang II can modulate baroreflex control of cardiac vagal drive. Although there is evidence that Ang II can modulate parasympathetic activity and has the potential to contribute to impaired baseline levels and impaired baroreflex control during hypertension, the exact central regions where Ang II acts need further investigation. The beneficial actions of angiotensin receptor blockers in hypertension may be mediated in part via actions on the parasympathetic nervous system. We highlight important unknown questions about the interaction between the RAAS and the parasympathetic nervous system and conclude that this remains an important area where future research is needed.

## 1. Introduction

The renin–angiotensin–aldosterone system (RAAS) is a complex, multifaceted system. Since the discovery of RAAS over a century ago, our understanding of it has expanded from one circulating hormone to multiple complementary and opposing enzymatic pathways that signal via autocrine and paracrine mechanisms [1,2]. Initially thought of as an endocrine system for regulating blood pressure, components of the RAAS pathway have now been identified across all organs [3,4] and likely modulate organ function.

To date, there has been extensive interest in the interactions between the RAAS and the autonomic nervous system. Most research has focused on the bidirectional complementary actions of the RAAS and the sympathetic nervous system, with less attention on the parasympathetic nervous system. The interactions between the RAAS system and sympathetic signalling in health and disease have been extensively studied and reviewed elsewhere [2,5,6,7]. Briefly, activating the RAAS pathways increases sympathetic neurotransmission centrally and at the autonomic ganglia, spinal cord, and end-organs [2,5]. The effects of the RAAS are predominantly mediated by angiotensin (Ang) II binding to cell-surface AT_1_ G_q_ protein-coupled receptors. In vascular smooth muscle cells, this results in inositol triphosphate (IP_3_)-mediated vasoconstriction, resulting in increased blood pressure. In addition, the actions of Ang II also lead to vasopressin release, oxidative stress, inflammation, and activation of central brain regions leading to sympathetic activation [6,8]. 

The second arm of the autonomic nervous system is the parasympathetic nervous system. The parasympathetic nervous system and vagal tone have long been branded as the ‘rest and digest’ arm of the autonomic nervous system—a simple pathway to offset the excitatory sympathetic nervous system and to maintain homeostasis. The parasympathetic nervous system regulates an extensive range of functions from blood pressure and heart rate to breathing and immune responses [9,10,11]. Increasing research into the parasympathetic nervous system is advancing our understanding of the nuances of how it works in both health and disease. RAAS is often stated to decrease parasympathetic nervous signalling [5,12], although direct evidence to support this, and the downstream signalling involved, are rarely provided. Here, we review the role of the RAAS on parasympathetic nerve function during normal physiology and potential dysregulation in cardiovascular disease, focusing on hypertension. We evaluate the clinical implications of these findings and comment on future directions for RAAS and the forgotten branch of the autonomic nervous system.

## 2. Autonomic Dysregulation and the Role of RAAS Signalling in Hypertension

### 2.1. The Problem of Hypertension

Hypertension is a leading preventable risk factor for all-cause mortality worldwide [13,14], and one of the most important prognostic indicators of cardiovascular disease [15,16]. Hypertension is defined as elevated blood pressure (BP) of systolic BP ≥ 140 mmHg and a diastolic BP ≥ 90 mmHg. It is estimated that over 30% of the adult population worldwide have hypertension [17]. The first anti-hypertensive medications were used clinically in the late 1940s and early 1950s [18]. Early agents included thiazide diuretics, which targeted blood volume to reduce blood pressure [18]. Since then, scientific research and clinical trials have significantly expanded our understanding of the aetiologies underlying hypertension. Increased sympathetic nervous system activity and reduced vagal tone are established characteristics of hypertension [19,20,21,22]. This dysregulation of the autonomic nervous system is correlated with progression of disease [23], is observed in those classed as borderline hypertensive [20], and has a genetic predisposition to developing hypertension [24]. Many currently prescribed therapeutics for the treatment of hypertension, including β-blockers and α-blockers, target the overactivity of the sympathetic nervous system [25]. Other main anti-hypertensive agents include diuretics; drugs that target the RASS, including angiotensin receptor blockers (ARBs) and angiotensin converting enzyme (ACE) inhibitors; and Ca^2+^ channel antagonists, which mediate vasodilation [26]. Although hypertension is preventable, an estimated 10–30% of hypertensive patients are treatment resistant [27], defined as BP remaining uncontrolled on four or more anti-hypertensive agents, including a diuretic [27]. At the same time, there is debate on the number of patients who are genuinely treatment resistant or those who are noncompliant with low adherence to their treatment regime [28], likely due to deleterious side effects of their medications. The global and personal burden hypertension presents for cardiovascular mortality shows the need for new therapeutic treatments.

### 2.2. Actions of Ang II in Hypertension

The cellular and molecular mechanism by which Ang II contributes to increased vasoconstriction and a rise in blood pressure have been extensively studied and form the backbone of many mainstream treatments of hypertension [29,30]. ARBs and ACE inhibitors were traditionally prescribed as anti-hypertensives to target ‘classical’ RAAS pathways to prevent IP_3_-mediated vasoconstriction and to inhibit sympathetic nerve activity, in part by increasing central neuronal nitric oxide synthase (nNOS) [31,32,33]. The central control of sympathetic nerve activity by Ang II has been extensively studied in rodent models [6,7,34,35,36,37]. Emerging evidence is looking into the role of RAAS in ‘atypical’ pathways beyond its regulation of vasoconstriction. Ang II-mediated increases in reactive oxygen species (ROS) lead to increased activity via NADPH oxidase 2 (Nox2) [38], increased sympathetic nerve activity [39,40], and the activation of p38 MAPK, regulating cell survival [41]. The Ang II/ROS pathway is thought to be the leading cause of centrally increased neuroinflammation and sympathetic outflow in rodent models of hypertension [6,39,40,42,43,44]. In cardiovascular disease, AT1Rs have been shown to regulate hypertrophy by coupling to JAK-STAT pathways [41]. Moreover, via RhoGEF (Rho guanine nucleotide exchange factors) and JNK, Ang II can promote extracellular matrix formation [41], neuron development, neurotransmitter release [45,46], and vasoconstriction [47], highlighting the complexities of RAAS signalling in pathology.

To date, the beneficial effects of ARBs and ACE inhibitors have been put down to alleviating vasoconstriction and sympathetically mediated symptoms. The reduction in parasympathetic nerve activity observed in hypertension, coupled with our knowledge of the beneficial effects of restoring vagal tone in cardiovascular diseases suggests that the role of Ang II in the regulation of the parasympathetic nervous system warrants further investigation. The next sections focus on the role of Ang II specifically on the parasympathetic nervous system.

## 3. Receptors of Ang II Are Found throughout the Parasympathetic Nervous System

**Figure 1:**
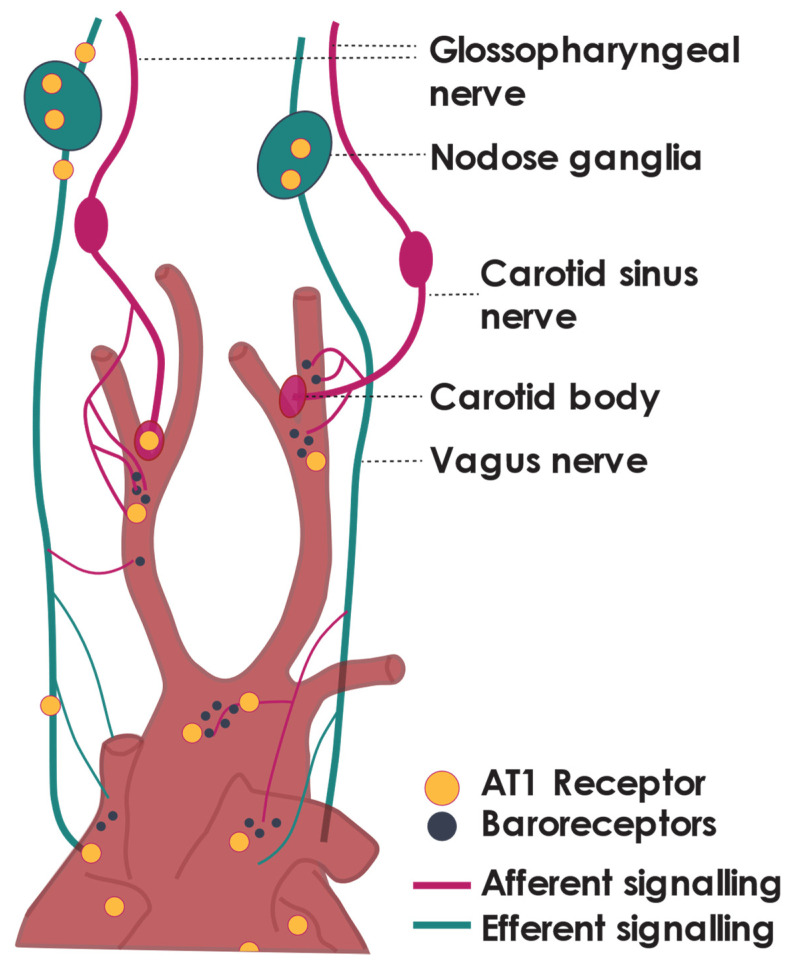
Sites of Ang II modulation of the cardiac baroreflex. AT1 receptor is marked with yellow circles. Baroreceptors are marked with dark grey circles.

Radiolabelled binding studies in the 1980s and more recent studies utilising AT_1_ receptor (AT1R) flox/flox mice have identified AT1Rs and Ang II binding sites in all major parasympathetic central integration regions. Ang receptors have been identified within the nodose ganglia, at presynaptic terminals, along the vagal trunk, and at the terminals of vagal afferent fibres in the heart [48,49] (Figure 1). Ang II receptors are also found in the nucleus of the solitary tract (NTS), where they are associated with vagal afferent terminals, and the dorsal motor nucleus (DMV) and nucleus ambiguous (NA), the sites of origin of vagal efferent neurons [48,49,50,51]. In line with early work investigating the interactions between RAAS and the sympathetic nervous system [52], current evidence for RAAS mediation of the parasympathetic tone focuses on an Ang II AT1R pathway. There is some additional evidence for Ang (1–12) and Ang (1–7) regulating parasympathetic tone [53,54] although work on this is limited.

In addition to central regions, AT1Rs on preganglionic nerve fibres provide a mechanism by which Ang II can directly modulate autonomic neurotransmission [55]. AT1Rs are present on preganglionic fibres of both sympathetic and parasympathetic nerves [5]. Vagal ligation distal to the nodose ganglia results in an accumulation of receptor binding sites proximal to the ligature, suggesting that Ang II receptors are transported centrally to vagal integration centres of the NTS from the nodose ganglia [48]. A lower proportion of Ang II binding sites also accumulated on the distal side of the ligature, indicating that Ang II receptors present in the cell bodies of the nodose ganglia may also be transported to the end organ terminals of the vagus [48]. These studies show that Ang II binding sites receptors undergo bidirectional transport along the vagus nerve. Experiments on isolated rat nodose ganglia have shown that Ang II (in addition to, Ang I and Ang III) can directly and concentration-dependently elicit depolarisation of the nodose ganglia [56]. These data suggest that Ang receptors are present along with multiple sites of the parasympathetic nervous system and therefore have the capacity to alter parasympathetic tone. While it remains unclear how Ang II would reach these receptors, the next section focuses on evidence that Ang II can alter vagal tone. 

## 4. Ang II Tonically Inhibits Cardiac Vagal Tone—Peripheral Actions

Several studies have suggested that cardiac parasympathetic tone is under tonic inhibition by Ang II [57,58,59]. Stimulation of the main vagal branch results in depolarisation-induced release of acetylcholine from postganglionic nerve fibres and reduces heart rate. Vagal nerve stimulation-induced bradycardia is potentiated by blocking AT1Rs with the antagonists losartan and KT3-671, indicating an essential physiological role for endogenous Ang II in modulating vagal tone. A potentiation of vagal-induced bradycardia is also seen after intravenous infusion of the ACE inhibitor captopril [58,60]. Interestingly, AT1R antagonists did not affect bradycardia induced by exogenous acetylcholine. This indicates that the site of tonic inhibition of vagal tone by Ang II appears to be preganglionic or at the level of the postganglionic neuron [58].

Moreover, intravenous infusion of Ang II attenuated the vagal nerve stimulation-induced bradycardia, and this inhibitory effect of Ang II on bradycardia is restored by the anti-hypertensive losartan. These results are supported by studies that measured the effect of vagal nerve stimulation on myocardial interstitial acetylcholine release using microdialysis probes in anaesthetised cats [61]. An intravenous infusion of Ang II suppressed vagal stimulation-induced acetylcholine release. This suppression of acetylcholine release was reversed by pre-treatment with intravenous losartan, pointing to a role for AT1R. However, the local infusion of losartan through a cardiac dialysis probe did not prevent the Ang II-mediated suppression of acetylcholine release [61]. These results suggest that the inhibitory action of Ang II on acetylcholine release is likely due to a presynaptic rather than postsynaptic action. However, how far the losartan can diffuse out of the dialysis probe to the vagal nerve terminals could not be determined so a role for the postsynaptic receptors cannot be ruled out. These actions of Ang II on vagal tone appear to be mediated via AT1Rs since no effect on vagal stimulation-mediated bradycardia is observed with an AT2R antagonist [57].

An intracellular pathway via which Ang II acts pre-ganglionically to inhibit acetylcholine release remains unknown. We know that AT1R can elicit the activation of a diverse range of second messenger pathways, dependent on which heterotrimeric G-protein the AT1R interacts with [62]. The work by Huang et al. suggested that the AT1A receptor subtype is expressed in the rat vagus and central integration regions [63]. AT1A receptors interact with many G-proteins and other non-G-proteins via the carboxyl-terminus [64]. It is worth noting that, while AT1A and AT1B receptor subtypes have been identified in rodents, only one AT1R subtype has been identified in people [7]. The AT1R downstream signalling that elicits reduced acetylcholine release is not known but is likely to involve pathways that reduce intracellular calcium or result in membrane hyperpolarisation. It is unlikely to be via the canonical G_q/11_–AT1R coupling, which signals via the inhibition of cAMP and the generation of ROS, respectively [65]. These studies indicate that this pathway may be an additional mode of action via which Ang II blockers may be beneficial [57,59]. In conclusion, these data taken together indicate that losartan and other clinically available AT1R blockers potentiate vagal tone. Given that the restoration of vagal tone can be beneficial, AT1R blockers should be considered for other conditions in which potentiating vagal tone would be beneficial.

## 5. Ang II Attenuates Cardiac Vagal Activity—Central Actions

### 5.1. Ang II within the Brain

While Ang receptors are present within central brain regions, it is unclear how circulating Ang II can access these receptors. Ang II cannot cross the blood–brain barrier under normal conditions. One possibility is the local production of Ang II, and indeed, the presence of Ang II synthesis and local tissue RAAS was first identified in canine brains in the 1970s [66]. Since then, local tissue RAASs have been identified and studied extensively in different tissues, including but not limited to the heart, blood vessels, brain, and reproductive and lymphatic tissues [2,35,67]. In the brain, Ang II synthesis and other aspects of the RAAS pathway have been identified in a wide variety of neuronal and nonneuronal cell types [6,68,69,70]. Central Ang II synthesis and its potential role in sympathetically mediated blood pressure regulation has been extensively studied and reviewed elsewhere [7,35]. The role of Ang II on central regulation by nonneuronal cells and their interacting influence on neuronal cells remain under active investigation [6,68,71]. To date, work has predominantly focused on the interacting influences of glial cells with neuroinflammation and increased sympathetic outflow [6,58,72]. How Ang II and nonneuronal cells types may modulate parasympathetic tone is currently unknown. It is important to note that care must be taken when interpreting studies that have determined the location of AT1Rs using antibodies, as the specificity of some AT1R labelling techniques have been questioned [73]. Despite this caveat, the general consensus is that Ang II receptors are present in central brain regions and have the potential to impact neuronal function once Ang II reaches these receptors.

While Ang II cannot cross the blood–brain barrier in normal conditions, an Ang II mediated disruption of the blood–brain barrier is seen in rodent models of hypertension and may play a role in addition to central synthesis of Ang II. Increased circulating Ang II in hypertension results in vascular inflammation and increased blood–brain permeability that facilitates access by Ang II to other brain regions [74,75]. Indeed, once the blood–brain barrier is disrupted, Ang can activate neurons past the blood–brain barrier [76]. This may suggest a pathway by which Ang II can enter the brain in conditions of high RAAS activation, such as hypertension and heart failure. Ang II may also influence central processes by signalling neuronal pathways from the circumventricular organs: regions with no tight blood–brain barrier, such as the subconical organ [77]. Others have suggested that Ang II may not need to enter the brain itself but can signal through a vascular AT1R-mediated release of nitric oxide, which then diffuses into the brain and the site of action [78]. The role of Ang II in regulating central sympathetic neurotransmission in hypertension has been extensively researched and reviewed elsewhere [79]. Here, we focus on the potential actions of Ang II on parasympathetic brain regions. 

### 5.2. Ang II in Central Brain Regions That Can Modulate Parasympathetic Nerve Activity

**Figure 2:**
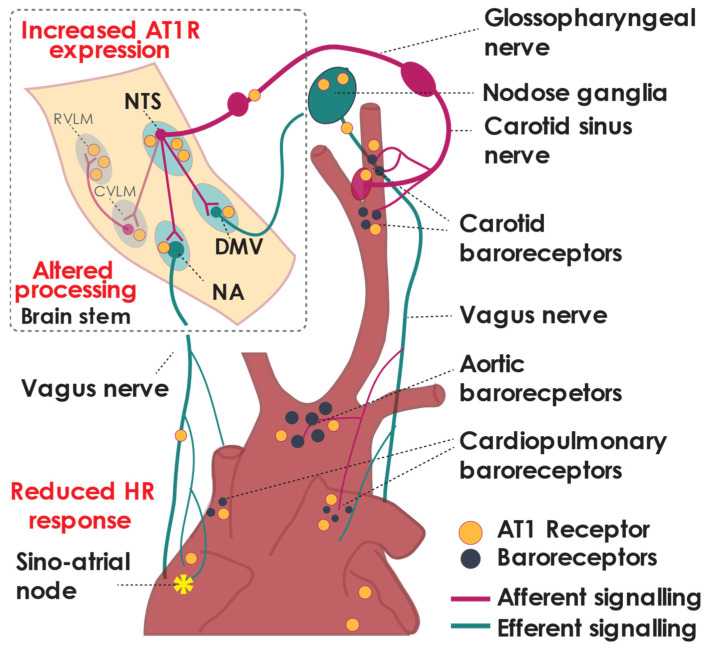
Baroreflex signally is altered in hypertension. Altered integration of the afferent baroreflex signal in the brain regions of the NTS and NA, affect heart rate response to baroreflex activation. NTS, nucleus tractus solitarii. DMV, dorsal motor nucleus of the vagus. NA, nucleus ambiguous. RVLM, rostral ventrolateral medulla. CVLM, caudal ventrolateral medulla.

Within central brain regions that can directly impact parasympathetic nerve activity, the RAAS modulation of parasympathetic signalling has predominantly been studied in the NTS and the DMV. Less is known about Ang II regulation in the nucleus ambiguous (NA). The NTS is the primary integration site of afferent nerve fibres. Afferent integration through the NTS modulates both sympathetic activity, via the caudal and then rostral ventrolateral medulla (CVLM and RVLM, respectively), and parasympathetic activity via the DMV and NA (Figure 2) [80,81]. Spinal and vagal afferent fibres converge on the NTS, including all fibres from the aortic depressor nerve and the carotid sinus nerve [82,83]. Signalling via the NTS has been proposed to be essential for blood pressure regulation, baroreflex control [82,84], and the maintenance of hypertension in hypertensive animal models [84]. The NA and DMV within the medulla oblongata receive input from the NTS to modulate efferent cardiac effector functions: the NA predominantly innervates the sino-atrial node (SAN) and modulates heart rate [85,86], whereas the DMV predominantly controls contractility [85,87]. 

To determine the role of central Ang II on central parasympathetic regulation, microinjections of Ang II has been previously used. Ang II injection in both the DMV and the NTS of rats produced both a fall in blood pressure and heart rate. Bilateral vagotomy before Ang II injection in the DMV or NTS attenuated the bradycardic response without affecting the fall in blood pressure [88], suggesting that Ang II AT1R within the NTS and DMV can augment vagal tone. However, no consistent change in blood pressure or heart rate was observed after Ang II injection into the DMV of canines [89]. Evidence for the role of endogenous Ang II within the NTS, particularly in cardiovascular disease, or conditions of high sympathetic drive comes from a series of experiments where AT1R antagonists are injected into the NTS of rats with atriovenous (A–V) shunts. Similar to the injection of Ang II, a decrease in heart rate and blood pressure is observed [90], concluding that these antagonists must block chronic activation by Ang II, which existed previously. It is difficult to reconcile these two sets of experiments given that both agonists and antagonists produced a similar response. It must be noted that the NTS contains multiple afferent nerve fibres in addition to parasympathetic afferents. These include but are not limited to visceral sensory afferent fibres that synapse at the dorsal root ganglia and innervate the NTS via the spinal column. The stimulation of visceral sensory fibres is known to increase the activity of sympathetic efferent nerve activity [91]. As such, the conflicting responses may be due to selective actions of Ang II on distinct sets of afferent neurons. More work is needed to define the precise role of Ang II in the NTS in response to vagal or spinal afferent nerve activation. 

A significant clinical benefit of AT1R antagonists in the treatment of hypertension is that they produce a drop in blood pressure without a reflex increase in heart rate. AT1R antagonism of Ang II centrally is postulated to reduce both sympathetic and parasympathetic outflow, producing this clinically beneficial effect. An increase in AT1R binding sites in both the NTS and RVLM of pre-hypertensive SHR rats has led to the conclusion that increased AT1R signalling in autonomic control centres of the brain is essential for the development of hypertension in some models [7,36,37]. No effect on blood pressure or heart rate was observed after the administration of an AT2R antagonist in rats with A–V shunt [90]. In contrast, increasing AT2R expression in the NTS and DMV of renovascular hypertensive rats attenuated their hypertension [92]. Given that AT2R has been identified in the NTS in some rat models [93], this highlights a potentially beneficial role of Ang II signal via AT2R in parasympathetic brain regions. Work on this is limited and the function of the AT2R within the NTS, NA, and DMV clearly warrants further investigation.

## 6. Ang II and Its Actions on the Baroreflex

### 6.1. Baroreflex Dysfunction in Hypertension

The arterial baroreflex is a homeostatic mechanism maintaining stable blood pressure. Arterial baroreceptors within the carotid sinus and aortic arch activate in response to increased vascular tension [94]; this signal travels via afferent nerve fibres of the carotid sinus nerve, synapsing within the nodose or petrosal ganglia [82] (Figure 1). The afferent information is integrated within the NTS and relayed via the NA and, to a lesser extent, the DMV [95] (Figure 2), activating efferent parasympathetic pathways resulting in reduced heart rate, sympathetic nerve activity, and systemic vascular resistance [96,97]. The role of baroreceptors in conditions where normal blood pressure cannot be maintained, such as hypertension, is an area of great scientific and commercial interest [98]. 

**Figure 3:**
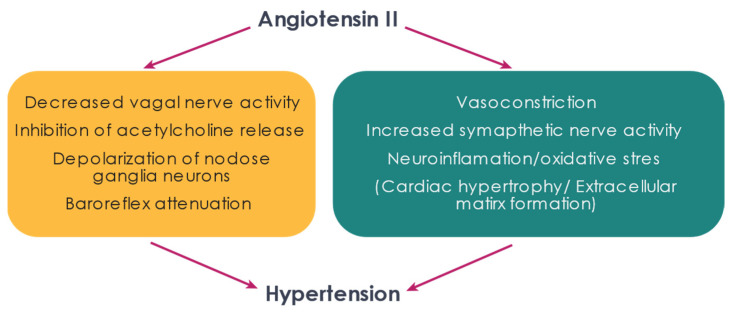
Ang II contributes to the generation of hypertension through actions on vasculature and the autonomic nervous system. Targeting these pathways may be therapeutically beneficial in the treatment of hypertension.

The baroreflex is significantly attenuated in hypertension (Figure 2 and Figure 3), as is the modulation of vagal tone. Impaired baroreflex function is a clinical prognostic indicator in patients with cardiovascular disease, including hypertension and heart failure [99,100]. Reduced baroreflex sensitivity can be used as an independent indicator of mortality and major adverse cardiovascular events in hypertensive patients [101,102] and is correlated with disease progression from borderline to established hypertension [103]. The prevalence of reduced baroreflex sensitivity in hypertension is observed even in normotensive individuals with a family history of hypertension [104]. Interestingly, while the baroreflex control of heart rate is reduced, the regulation of muscle sympathetic nerve activity is not [105,106], although resting sympathetic nerve activity is higher in hypertensive patients [106]. Animal studies have indicated that the baroreflex control of directly recorded cardiac sympathetic nerve activity is preserved in hypertension [107], suggesting that the impaired baroreflex regulation of heart rate is due to impaired vagal control of the heart. It is thought that, during hypertension, central control of the baroreflex ‘resets’ to activate at a higher blood pressure, although the mechanisms behind this are unknown [108,109]. 

The prominence of baroreflex dysregulation in hypertension and the observed clinical benefits when the baroreflex is increased have led to great interest in developing new treatments to target the baroreflex. Current treatments aiming to restore baroreflex function include device-based therapies designed to electrically stimulate the carotid sinus nerve in response to a sensed increase in blood pressure [98]; exercise training [110]; and slow breathing exercises, which increase vagal tone [100,111]. 

### 6.2. Actions of Ang II on the Baroreflex

Ang II within multiple brain regions has been shown to increase sympathetic nerve activity [32,112]. This sympatho-excitatory response elicited by Ang II is, in part, caused by reduced activation of the baroreflex. Ang II inhibits baroreflex signalling by resetting the set point at which systolic blood pressure triggers carotid sinus afferent nerve activity and decreases baroreflex sensitivity; this has been observed across multiple animal models and humans [12,113,114,115,116]. Resetting the baroreflex compounds the hypertensive effects observed with Ang II as it blunts the body’s homeostatic mechanism to reduce blood pressure. Multiple sites through the baroreflex arc, including the afferents, central regions, and efferent nerves, provide sites where the RAAS pathway may modulate this reflex. While Ang II receptors exist throughout the baroreflex arc (Figure 1 and Figure 2), their functional importance at each site is not fully understood [117]. While the number of studies is limited, we focus on the actions of Ang II at the afferent nerve endings as well as central regions of the baroreflex pathway. 

A study in anesthetised cats examined what happened to carotid sinus nerve activity in response to intravenous or intra-carotid Ang II; the authors observed that the increase in carotid sinus nerve activity observed with Ang II was no different to that seen when blood pressure was increased with phenylephrine [118]. These data suggested that Ang II does not alter baroreflex sensitivity at the level of the afferent nerves, although intra-carotid Ang II administration was used as a method to increase central Ang II. It is now well documented that, in most conditions, Ang II cannot cross the blood–brain barrier so this suggests that the actions of Ang II may not be at the baroreceptor afferent nerve endings. This is in agreement with a second study that used direct nerve fibre recordings from the afferent carotid sinus nerve and the efferent vagal nerve. The authors studied the chronic infusion of Ang II and found no alteration in baroreceptor firing from the carotid sinus nerve in these hypertensive animals [119]. However, these authors observed a decrease in vagal efferent nerve firing [119]. With the chronic infusion of Ang II, the circulating Ang II can access the circumventricular organs and this may be one site of action of Ang II. The area postrema, within the circumventricular organs, has been shown to mediate Ang II resetting of the baroreflex within both intact and area postrema lesioned animal models [120,121]. 

Intravenous (iv) or intracerebroventricular (icv) infusion of Ang II is often used as a way to induce neurogenic hypertension in animal models [122,123]. Ang II-mediated hypertension results in the activation of neurons in the NTS as well as sympathetic central regions including the caudal ventrolateral medulla and rostral ventrolateral medulla [123] and a reduction in baroreflex sensitivity [124] (Figure 2). The injection of Ang II directly into the NTS [125,126,127] and lesioning the NTS in animal models of hypertension abolished the carotid baroreflex response, highlighting the importance of central integration in this process [84]. More recent studies utilising targeted microinjection techniques have shown that Ang II modulation of the baroreflex is at least in part due to an AT1R-mediated pathway in the NTS [126,128]. ICV infusion of Ang II in c57BL/6 mice led to increased levels of NADPH oxidase-generated by superoxide in the NTS and reduced cardiac vagal baroreflex sensitivity [122]. The increased generation of reactive oxygen species by Ang II, resulting in disrupted signalling between the NTS and NA in relation to baroreflex control in hypertension, warrants further investigation [122]. In addition to the NTS, Ang AT1R within the CVLM can also reduce baroreflex sensitivity postulated to be via a cardiac vagal action [129]. 

Microinjections of ACE inhibitors into the NTS can restore baroreflex sensitivity towards control levels and therefore may be therapeutically beneficial [130]. Moreover, increased expression of AT2R within the parasympathetic brain regions reversed baroreflex dysfunction in a renovascular hypertensive rat [92]. These results suggest the role of Ang II in baroreflex sensitivity in hypertension may not be as simple as Ang II binding to AT1Rs and that alternate RAAS pathways may be targeted in hypertension to positively modulate this pathway [130]. These studies taken together suggest that the actions of Ang II on the baroreflex is mediated via central actions to reduce parasympathetic efferent outflow rather than an afferent mechanism at the level of the carotid sinus. 

## 7. Beyond Ang II

The Ang (1–7) branch of the RAAS pathway is often thought to counteract the Ang II arm of the classical RAAS pathway. While there is much recent interest in these “non-traditional” arms, there is little conclusive evidence for additional aspects of the RAAS pathway, beyond Ang II, in the modulation of vagal tone. Ang (1–7), ACE2, and MasR have been proposed to increase parasympathetic tone [5,131]. Most of this work has looked at baroreflex modulation and other surrogates of autonomic drives, such as reduced blood pressure in hypertensive models. Therefore, it is difficult to establish whether the actions of Ang II are via reduced sympathetic nerve activity or increased parasympathetic nerve activity. The central infusion of Ang (1–7) in conscious rabbits enhances baroreflex gain via a proposed mechanism of increased vagal activity [132]. Enhanced baroreflex gain was also observed following Ang (1–7) microinjection into the NTS of normotensive rats, although this result was blunted in hypertensive rats, which showed reduced sensitivity to Ang (1–7) [133]. The overexpression of Syn-hACE2 (human ACE2 under a synapsin promotor) in murine brains attenuated Ang II blunting of the baroreflex sensitivity [134]. These results corroborate the increased baroreflex sensitivity seen with the direct NTS overexpression of ACE2 in a hypertensive rat [135], indicating that there may be a beneficial role for central ACE2 in parasympathetic and baroreflex regulation.

Ang (1–12) can be converted by ACE or chymase into Ang II or via Ang (1–8) to Ang (1–7). When Ang (1–12) is injected into the NTS of rats, it activates baroreflex afferent nerves, producing a reduction in mean arterial pressure via sympathetic activation and a reduction in heart rate by parasympathetic activation. These responses were shown to be due to the conversion of Ang (1–12) to Ang II by both ACE and chymase, with no Ang (1–7) [136]. Knockout of the pro-renin receptor of the mouse paraventricular nucleus (PVN) can also increase parasympathetic tone, which was differential to the decreased sympathetic tone observed with PVN pro-renin receptor knockout [137]. While there are not enough studies to provide a complete picture of how these non-traditional arms of the RAAS system alter vagal tone, the evidence to date suggests that there is clearly a role and that further studies are needed.

## 8. Perspectives and Future Directions

Our understanding of the role of the RAAS on vagal tone is mainly confined to understanding the role of the RAAS on baroreflex sensitivity. While there is evidence that the RAAS can modulate vagal tone, there has been little concerted effort to understand the pathways or sites of action where the RAAS pathways may interact with parasympathetic pathways. While there is evidence that Ang II reduces vagal tone to the heart, there is even less published work investigating the role of the extended RAAS pathway, including Ang (1–7), ACE2, pro-renin, and MasR in parasympathetic control. The expanding intricacies of RAAS signalling concerning sympathetic neurotransmission suggest that a lot more about RAAS regulation of vagal control is still to be discovered.

Clinical alterations in autonomic neurotransmission worsen disease progression. Restoring autonomic imbalance can improve patient outcomes, and current device-based therapies in cardiology stimulate the baroreflex or vagal nerve to restore autonomic balance in heart failure [98]. Therefore, a better understanding of the role of Ang II on parasympathetic regulation will give us a better understanding of how any treatment, pharmacological or device-based, may influence the parasympathetic nervous system. In addition, a better understanding of novel treatments that target the RAAS pathway could produce beneficial effects in the future.

Assessing the specific role that Ang II and the RAAS pathway have on parasympathetic function is limited by the complexities of studying the vagal nerve. The vagus is a mixed nerve bundle containing both afferent and efferent fibres, and stimulating the main vagal branch does not differentiate between afferent and efferent fibres. There is some ongoing work in this area to establish novel vagal branch stimulation protocols to selectively stimulate only a subset of fibres [138,139]. The advancement of neuron-specific mouse models; viral gene transfer; and new technologies, including optogenetic and chemogenomic stimulation, has allowed for more specific study within these pathways [140]. Another limitation to studying the role of Ang II in parasympathetic neurotransmission is the lack of reliable and reproducible ‘parasympathetic’ cell lines, although recent progress may change this in the future [141]. A stepwise neurotropic chemical induction method of human pluripotent stem cells developed by Takayma et al. can be used to induce both sympathetic-like and parasympathetic-like autonomic nervous system neurons [141]. Work has been published on modifying this technique to study sympathetic neurons; however, its use for differentiation of parasympathetic cells is still untested [142].

The current antihypertensives that target the RAAS pathway are AT1R antagonist and ACE inhibitors. The role of AT1R-mediated reduction in cardiac acetylcholine release warrants further investigation to establish if current clinically approved AT1R antagonists could provide a beneficial effect through this pathway. Ang (1–7) is emerging as a major endogenous product of the RAAS system found in the brain across animal models [131]. It has been suggested that some of the actions originally ascribed to Ang II may actually be regulated by other Ang peptides, such as Ang (1–7), which is a promising candidate for the central regulation of vagal tone [131]. Other candidates for future targeting include AT2R since improvements in baroreflex sensitivity have been shown with the overexpression of AT2R in central brain regions [143]. The role of Ang II-mediated generation of reactive oxygen species and its role in parasympathetic regulation is an additional exciting development in this field [122]. Expanding our understanding of the RAAS pathway’s roles on vagal regulation, both centrally and peripherally, helps us understand how these two pathways interact in disease and correct conditions of reduced vagal tone. 

## Data Availability

Not applicable.
